# Influence of Coating and Size of Magnetic Nanoparticles on Cellular Uptake for In Vitro MRI

**DOI:** 10.3390/nano11112888

**Published:** 2021-10-28

**Authors:** Belén Cortés-Llanos, Sandra M. Ocampo, Leonor de la Cueva, Gabriel F. Calvo, Juan Belmonte-Beitia, Lucas Pérez, Gorka Salas, Ángel Ayuso-Sacido

**Affiliations:** 1 IMDEA Nanoscience, Ciudad Universitaria de Cantoblanco, 28049 Madrid, Spain; belencortess@ucm.es (B.C.-L.); samioca75@gmail.com (S.M.O.); ldelacueva@euro-funding.com (L.d.l.C.); lucas.perez@ucm.es (L.P.); 2 Department of Bioengineering, University of Washington, Seattle, WA 98195, USA; 3 Department of Materials Physics, Complutense University of Madrid, 28040 Madrid, Spain; 4 MOLAB-Mathematical Oncology Laboratory, Department of Mathematics, Universidad de Castilla-La Mancha, 13071 Ciudad Real, Spain; gabriel.fernandez@uclm.es (G.F.C.); juan.belmonte@uclm.es (J.B.-B.); 5 Brain Tumor Laboratory, Fundación Vithas, Grupo Hospitales Vithas, 28043 Madrid, Spain; 6 Faculty of Experimental Sciences, Universidad Francisco de Vitoria, 28223 Madrid, Spain; 7 Faculty of Medicine, Universidad Francisco de Vitoria, 28223 Madrid, Spain

**Keywords:** iron oxide nanoparticles, colloidal properties, cellular uptake, magnetic resonance imaging

## Abstract

Iron oxide nanoparticles (IONPs) are suitable materials for contrast enhancement in magnetic resonance imaging (MRI). Their potential clinical applications range from diagnosis to therapy and follow-up treatments. However, a deeper understanding of the interaction between IONPs, culture media and cells is necessary for expanding the application of this technology to different types of cancer therapies. To achieve new insights of these interactions, a set of IONPs were prepared with the same inorganic core and five distinct coatings, to study their aggregation and interactions in different physiological media, as well as their cell labelling efficiency. Then, a second set of IONPs, with six different core sizes and the same coating, were used to study how the core size affects cell labelling and MRI in vitro. Here, IONPs suspended in biological media experience a partial removal of the coating and adhesion of molecules. The FBS concentration alters the labelling of all types of IONPs and hydrodynamic sizes ≥ 300 nm provide the greatest labelling using the centrifugation-mediated internalization (CMI). The best contrast for MRI results requires a core size range between 12–14 nm coated with dimercaptosuccinic acid (DMSA) producing *R*_2_^*^ values of 393.7 s^−1^ and 428.3 s^−1^, respectively. These findings will help to bring IONPs as negative contrast agents into clinical settings.

## 1. Introduction

In vitro and in vivo nanoparticle (NP)-cell labelling, and tracking have become very promising techniques in biomedicine due to their clinical applications, spanning from regenerative medicine to the diagnosis and treatment of several diseases [1,2,3]. Among other nanomaterials, superparamagnetic iron oxide nanoparticles (IONPs) attract special attention because they can be remotely actuated through the application of magnetic fields that can easily penetrate in tissues [4,5]. IONPs are already used for the treatment of different tumors, mainly prostate [6] and glioblastoma [7], as well as contrast agents in magnetic resonance imaging (MRI) or magnetic particle imaging (MPI) and, in general, are considered to be relatively safe [8]. Their use has been approved in humans within clinical assay context to test enhancement of MRI diagnostic for different type of cancers [9,10,11,12,13,14,15,16], Multiple Sclerosis [17,18], myocardial infarction [19,20], vascular Inflammation in migraine without Aura [21], and inflammatory cell labelling and tracking with MRI after myocardial infarction [22]. However, increasing the intracellular internalization in a significantly faster and controlled way, both in vitro and in vivo, remains one of the major challenges to fully translate and exploit their capabilities in the clinical practice [23,24,25]. A handy approach for enhancing the uptake of nanoparticles (NPs) by cells in very short times is the centrifugation-mediated internalization (CMI) [26]. CMI has been applied for labelling living cells with high efficiency in a few minutes. Recently, experimental results using the CMI and other classical methods, together with numerical simulations of different transport mathematical models, provided evidence that the colloidal properties of IONPs have a strong influence on their internalization by cells, and that the coating of IONPs plays an important role in this process [27].

IONPs are usually composed of a magnetic core of iron oxide, typically magnetite (Fe_3_O_4_) or maghemite (γ-Fe_2_O_3_), and a hydrophilic coating to enhance their stability in aqueous suspension and their biocompatibility [28,29]. The magnetic properties of IONPs are size-dependent, so their behavior as MRI contrast agents depends strongly on their size [30]. IONPs are often employed for *T*_2_^*^-weighted MRI, and their contrast efficiency tend to increase with the size of the magnetic core [31]. IONPs in physiological media are usually aggregated and the characteristics of that aggregation, including, the aggregate size, also have an impact on the MRI contrast enhancement of IONPs [32]. Aggregation is common when IONPs are dispersed in liquid media and is almost unavoidable in physiological media [33]. It is influenced by the core size and, especially, by the surface coating and the dispersion media [34]. Therefore, the IONPs’ core size and coating are key features to be considered for cell labelling and tracking by MRI. 

Because of this, we have studied the influence of the core size and the coating of IONPs on the cell labelling efficiency by the CMI method, and on the MRI contrast enhancement in cell cultures. Firstly, we fixed the NPs core size and used five different coatings to assess what is the influence of the coating on the stability of IONPs in different cell culture media, as well as their interaction and aggregation with the media and on their internalization through the CMI method. Once we observed which was the coating presenting the higher labelling efficiency with good viability, we decided to fix the coating and change the core size. Then, IONPs with the same coating and six distinct core sizes were used to quantify the influence of the core size in the cell internalization and MRI. This study will open a new source of information regarding IONPs characterization to improve the labelling for MRI contrast enhancement. 

## 2. Materials and Methods

### 2.1. Materials

Iron(III) chloride (27% aqueous solution, with density 1.26 kg/L, VWR, Radnor, PA, USA), iron(II) chloride tetrahydrate (≥99.0%, Fluka, Charlotte, NC, USA), iron(III) acetylacetonate (97%, Sigma-Aldrich, St. Louis, MO, USA), ammonium hydroxide solution (25% in water, Fluka), iron(III) nitrate nonahydrate (≥98.0%, Fluka), sodium oleate (≥82%, Sigma-Aldrich, St. Louis, MO, USA), oleic acid (90%, Aldrich), oleylamine (70%, Aldrich), 1,2-dodecanediol (90%, Aldrich), 1-octadecene (90%, Aldrich), n-hexane (99%, Scharlau, Barcelona, Spain), toluene (99.8%, Sigma-Aldrich), dimethyl sulfoxide (>99.5%, Sigma-Aldrich) and ethanol (96%, Panreac, Barcelona, Spain), *meso*-2,3-dimercaptosuccinic acid (DMSA, 98% Aldrich), dextran (from *Leuconostoc* spp., Mr~40,000, Sigma-Aldrich, St. Louis, MO, USA), carboxymethyl-dextran sodium salt (10,000–20,000 Dalton, Sigma), diethylaminoethyl-dextran (average mol wt 40,000, Sigma), meso-2,3-dimercaptosuccinic acid (~98%, Aldrich), and (3-aminopropyl) triethoxysilane (98%, Alfa Aesar, Ward Hill, MA, USA) were used for the synthesis of IONPs. Dialysis tubing cellulose membranes were purchased from Sigma and washed before use. Phosphate buffered saline solution (PBS, 1x), Dulbecco’s modified eagle’s medium (DMEM), fetal bovine serum (FBS), L-glutamine, fungizone and antibiotics were purchased from GIBCO (Waltham, MA, USA). For the viability, the Resazurin dye was purchased from Sigma-Aldrich. Paraformaldehyde, nitric acid, hydrochloric acid and agarose were purchased from Sigma-Aldrich. For Prussian blue staining, potassium ferrocyanide (4%, Sigma-Aldrich), hydrochloric acid (4% Sigma-Aldrich), neutral red (0.5%, Panreac Quimica S.L.U, Barcelona, Spain), coverslips (GmbH & Co. KG, Maienfeld, Switzerland) and DePeX (SERVA Electrophoresis GmbH, Heidelberg, Germany).

### 2.2. IONPs Synthesis and Characterization

IONPs with the same core and different coatings were synthesized by the coprecipitation method, following the Massart’s method with slight modifications [35]. A total of 43 mL of ferric chloride 27% aqueous solution (0.090 mol) was diluted with 400 mL of water in a 1 L-beaker equipped with a magnetic stirrer. The solution was vigorously stirred (1200 rpm) and FeCl_2_∙4H_2_O (10.8 g, 0.054 mol) added. The orange solution was again diluted with 100 mL of water before slowly adding (addition time: 4 min) an aqueous solution of NH_4_OH 25% (75 mL). The solution turned black immediately due to the formation of magnetite (Fe_3_O_4_) NPs. After 5 min, the reaction mixture was heated until 90 °C and kept 3 h at that temperature. The reaction was allowed to cool down to room temperature and the black solid separated from the liquid with the help of a magnet (magnetic decantation). The solid was washed 3 times with water (3 times, 300 mL), stirred for 15 min and separated from the liquid by magnetic decantation.

Subsequently, the sample was subjected to acid treatment, as previously described [36], in order to oxidize Fe_3_O_4_ to γ-Fe_2_O_3_ and provide stability against oxidation [37]. A reddish-brown solid was obtained and redispersed in water (135 mL) for long-term storage (4 °C). The Fe concentration of this stock dispersion was determined by ICP-OES (70 mg/mL). The percentage yield was based on Fe measured by ICP and reached 70%.

#### 2.2.1. Dextran Coating of IONPs (NP-D)

A solution of NaOH (50 mg, 1.25 mmol) in water (0.5 mL) was added to 2 mL of the stock dispersion of IONPs. The dispersion contained 200 mg of γ-Fe_2_O_3_ (140 mg of Fe based on ICP) and is 0.5 M in NaOH. A solution of dextran (200 mg) in water (2.5 mL) was then added and the mixture sonicated for 10 h. Finally, the NPs were dialyzed for 3 days to remove impurities [38]. Yield = 86%.

#### 2.2.2. Diethylaminoethyl-dextran Coating of IONPs (NP-AD)

A total of 2 mL (200 mg of γ-Fe_2_O_3_) of the stock dispersion of IONPs were diluted with a solution of NaOH (50 mg, 1.25 mmol) in 16 mL of water to give a dispersion with pH = 12 that was then slowly added to another solution of diethylaminoethyl-dextran (200 mg) in 12 mL of water. The mixture was sonicated for 10 h and finally dialyzed for 3 days to remove impurities [39]. Yield = 79%.

#### 2.2.3. Carboxymethyl-dextran Coating of IONPs (NP-CMD)

A solution of carboxymethyl-dextran (200 mg) in water (2.5 mL) was added to 2 mL (200 mg of γ-Fe_2_O_3_) of the stock dispersion of NPs previously diluted with 0.5 mL of water. Then HNO_3_ (65%) was carefully added until pH = 3, the mixture sonicated for 10 h and then dialyzed for 3 days [40].

#### 2.2.4. (3-Aminopropyl) Triethoxysilane Coating of IONPs (NP-APS)

A total of 4.2 mL (410 mg of γ-Fe_2_O_3_) of the stock dispersion of IONPs were diluted with 9.8 mL of water and 14 mL of methanol. Then, under vigorous magnetic stirring, APS (1.71 mL, 7.3 mmol) was slowly added (addition time: 2 min). After 20 h, methanol was removed in a rotary evaporator and the resulting aqueous dispersion dialyzed for 5 days [41]. Yield = 82%.

#### 2.2.5. Dimercaptosuccinic Acid Coating of IONPs (NP-DMSA)

DMSA (10 mg, 0.055 mmol) was added to 0.910 mL (86 mg of γ-Fe_2_O_3_) of the stock dispersion of IONPs, previously diluted with water to a volume of 20 mL, and the mixture sonicated for 2 h. Then, KOH 1 M was added to reach pH = 11 and the sample dialyzed in water for 4 days [41]. Yield = 69%.

IONPs for comparing the different core sizes with the same coating were synthesized by thermal decomposition in 1-octadecene, following the method reported by Park et al. [42,43] with some modifications [44,45]. NPs of 7 nm were synthesized using Fe(acac)_3_ (5 mmol) as iron precursor, and with oleic acid (15 mmol), oleylamine (15 mmol) and 1,2-dodecanediol (25 mmol) as surfactants for the high temperature decomposition in 1-octadecene (50 mL). IONPs with larger sizes (12, 14, 18, 23, 33 nm) were synthesized using an iron(III)-oleate precursor (10 mmol) thermally decomposed at reflux in 1-octadecene (100 mL) in the presence variable amounts of oleic acid and different heating rates for the different core sizes. The so-obtained hydrophobic IONPs were coated with DMSA and transferred to aqueous suspension [44].

### 2.3. IONPs Characterization

#### 2.3.1. Colloidal Characterization

Hydrodynamic sizes *D_hyd_* in this work refer to the Z-average value measured by dynamic light scattering (DLS) from dilute suspensions of the samples in water at pH 7.4 or in Dulbecco’s modified eagle’s medium (DMEM) or DMEM supplemented with 10% fetal bovine serum (FBS) when specified, in a standard cuvette, using a Zetasizer Nano ZS device (Malvern Panalytical, Worcestershire, UK). The energy source was a laser emitting red light (633 nm), with an angle of 173° between the sample and the detector. Surface charges were evaluated by measuring the zeta-potential ζ values of samples diluted in aqueous KNO_3_ 0.01 M (or in DMEM or DMEM supplemented with 10% of FBS), with the same Zetasizer Nano ZS device.

#### 2.3.2. Thermogravimetric Analysis

The percentage weight of the organic coating was obtained by thermogravimetric/differential thermal analysis (TGA/DTA) carried out in a TA Instruments (New Castle, DE, USA) TGA 500 apparatus, with a heating rate of 10 °C/min from room temperature to 1000 °C in air atmosphere. For this analysis, aqueous samples were lyophilized to obtain the corresponding powders. TGA after incubation in different cell culture media (DMEM and DMEM + 10% FBS) and FBS, was performed using the following protocol: 1 mL of IONPs dispersion in biological medium (Fe concentration = 1 mg/mL) was mechanically stirred during 30 min. Then, the sample was centrifuged for 10 min at 13,000 rpm and the supernatant was removed. After that, the IONPs were redispersed in 1 mL of the same biological medium by slight sonication, until a homogeneous dispersion was obtained, and mechanically stirred for 30 min. The sample was again centrifuged (10 min at 1300 rpm), the supernatant removed and the residue of IONPs redispersed again in 1 mL of the biological medium. After 4 h of incubation under mechanical stirring, the IONPs were sedimented by centrifugation (10 min at 13,000 rpm), the supernatant discarded, and the resulting residue allowed to dry at air prior to the TGA analysis.

#### 2.3.3. Inductively Coupled Plasma (ICP)

The Fe content in the samples was determined by inductively coupled plasma-optical emission spectrometry (ICP-OES, PerkinElmer Optima 2100 DV ICP, Waltham, MA, USA) after dissolving the samples in HNO_3_:HCl 1:3 mixtures and diluting them with Millipore water.

#### 2.3.4. Transmission Electron Microscopy (TEM)

Particle size and shape were examined by TEM in a JEOL JEM 1010 microscope operating at 100 kV. Samples were prepared by placing one drop of a dilute suspension onto a carbon-coated copper grid and drying the drop with paper after two minutes. The size distributions were determined through manual analysis of ensembles of over 300 particles found in randomly selected areas of the enlarged micrographs, with Image Tool 3.00 software (UTHSCSA) to obtain the mean size and standard deviation.

#### 2.3.5. X-ray Powder Diffraction (XRD)

The X-ray powder diffractogram was recorded in the 2θ range from 0° to 80°, in a Siemens D-5000 powder diffractometer using Cu Kα radiation (λ_κα_ = 1.54 Å).

#### 2.3.6. Vibrating Sample Magnetometer (VSM)

The magnetic characterization was carried out in a vibrating sample magnetometer at room temperature (Lakeshore model 7410, Westerville, OH, USA) by first saturating the sample in a field of 2 T. Saturation magnetization (M_S_), expressed in emu per g of γ-Fe_2_O_3_ was evaluated by extrapolating the experimental results obtained in the high field range, where the magnetization linearly increases with 1/H, to infinite H (1/H = 0).

### 2.4. IONPs Internalization by Cells

#### 2.4.1. Cell Culture

U373 glioblastoma cell line was obtained from the American Type Culture Collections (Manassas, VA, USA). The cell line was grown in DMEM supplemented with 10% of FBS, 2 mM of L-glutamine, 1 μg/mL of fungizone, 100 μg/mL streptomycin and 100 unit of penicillin per ml (GIBCO). The cell line was maintained at 37 °C in a humidified atmosphere of 95% air and 5% CO_2_.

#### 2.4.2. IONPs Sterilization

To study IONPs uptake by cells, they were sonicated for 5 min before the incubations. Then, different sterilization procedures were performed: (i) the IONPs were mixed with DMEM (+10% FBS or without FBS) and filtered with a 0.22 μm Millex-GP filter (Merck-Millipore Darmstadt, Germany), or (ii) IONPs were sterilized by UV for 1 h and then mixed with DMEM (+10% FBS or without FBS).

#### 2.4.3. IONPs Uptake

CMI has been used to study the IONPs uptake. First, 5 × 10^4^ glioblastoma cells were plated in tubes of 15 mL and centrifuged at 1000 rpm for 5 min. Then, the supernatant was removed and 500 μL of IONPs solution at 25–125 μg Fe/mL concentration were mixed in DMEM and DMEM with 10% FBS. Depending on the proportion of FBS supplied to DMEM, 0% FBS or 10% FBS, the solution was centrifuged at 1500 rpm for 1 min or 5 min, respectively. Immediately after, the medium was exchanged with fresh medium and cells were plated in a 24 well plate and maintained at 37 °C for 24 h.

#### 2.4.4. Cell Viability

The cytotoxicity measurements were performed using the Resazurin dye (Sigma-Aldrich). This assay is used to measure cell viability and proliferation. After CMI method, the medium was replaced with 600 mL of fresh medium that contained 10% Resazurin dye. The samples were incubated at 37 °C for 3 h. Then, the amount of reduced Resazurin was determined by measuring the absorbance of the sample using a UV-visible spectrophotometer (Sinergy H4 microplate reader, Biotek, Winooski, VT, USA) at 570 nm of wavelength excitation and 600 nm of emission. For the negative control, 600 mL of medium with 10% of Resazurin dye was added to empty wells. After absorbance measurement, the medium was exchanged with fresh medium and the cells were incubated at 37 °C for 24 h. The viability of the cells was represented as the absorption percentage of treated cells normalized by control cells. All the samples were performed by triplicate.

#### 2.4.5. Labelling Efficiency

The labelling efficiency was calculated by counting the number of Prussian blue-stained and unstained labeled cells [26]. Six random optic fields from each sample were taken by light microscope (Leica DMI3000B, Leica Microsystems, Wetzlar, Germany). The estimation of the percentage was determined by the number of blue-stained cells divided by the total cell number per field.

#### 2.4.6. Scanning Electron Microscopy (SEM)

SEM images were made in a JEOL, JSM-6400 in order to observe the IONPs distribution on cells. After CMI method, the cells were washed twice with PBS and fixed with 4% paraformaldehyde and glutaraldehyde solution for 30 min. Then, a dehydration process was performed at different acetone concentrations (40%, 60%, 80% and 100%) for 5 min. The sample was washed twice for each dehydration step. Finally, the samples were dehydrated using the critical point procedure and coated with gold metal to make their surfaces electrically conductive. 

#### 2.4.7. Intracellular IONPs Quantification after CMI Method

After the CMI method using 75 µg/mL of initial IONPs, the media was removed and 500 µL of fresh DMEM was added. Then, a solution of nitric acid and hydrochloric acid (1:1) was added to the initial volume. The final solution, cells and uptake IONPs, was resuspended and sonicated for 30 min at 40 °C. After this step, 4 mL of water MQ was added to achieve the original 5 mL volume of the CMI method. From this solution, 2.5 mL was taken and mixed with a solution of Prussian blue, ferrocyanide and hydrochloric acid (1:1). After 15 min, 100 µL was added to a 96 well-plate (quadruplicate). The absorbance was read at 690 nm using a Zetasizer Nano ZS device (Malvern Panaytical, Worcestershire, UK). The concentration values were calculated from a calibration curve. This curve was calculated using four different initial concentrations of IONP12 10, 25, 50 and 100 µg/mL and following the same protocol as above.

#### 2.4.8. MRI Measurements

After carrying out the CMI method, labeled cells (5 × 10^4^) were fixed with PFA 4% for 20 min at room temperature. Afterward, the cells were washed twice with PBS and resuspended in 200 μL of 1% agarose (50 °C) in 96 well plates excluding air bubbles. The samples were then scanned and *T*_1_, *T*_2_, and *T*_2_^*^ weighted images were acquired with a Bruker Pharmascan 7 Tesla (16 cm). From all samples, 1 slice thickness of 1.5 mm was acquired on coronal orientation (FOV 3 cm). For *T*_1_ maps, RARE (rapid acquisition with relaxation enhancement) sequenced was used with the following parameters, TR: 70–6000 ms, TE: 12.6 ms; average: 1; acquisition matrix 128 × 128 (234 µm spatial resolution). *T*_2_ maps were based on the acquisition of 50 pondered images on *T*_2_ by MSME (multi-slice multi-echo) sequence with different eco times. The sequence parameters were the following ones, TR: 5000 ms, TE: 12–600 ms, 50 Ecos number, 1 average, acquisition matrix 128 × 128 (234 µm spatial resolution). For *T*_2_^*^ maps were obtained 10 pondered images on *T*_2_^*^ by MGE (multi-gradient echo) sequence with different eco times and these parameters, TR: 300 ms, TE: 78–40.67 ms, 10 eco numbers, 8 averages, acquisition matrix 256 × 256 (125 µm spatial resolution).

### 2.5. Statistical Analysis

The data were analyzed by GraphPad Prism version 6.0. All labelling efficiency samples were compared using a one-way ANOVA indicating significant differences when * *p* < 0.05, ** *p* < 0.01, *** *p* < 0.001. Unless otherwise noted, measurements are reported as the mean ± standard deviation.

## 3. Results and Discussion

### 3.1. Synthesis, Characterization and Colloidal Properties of IONPs with the Same Core and Different Coatings

**Figure 1:**
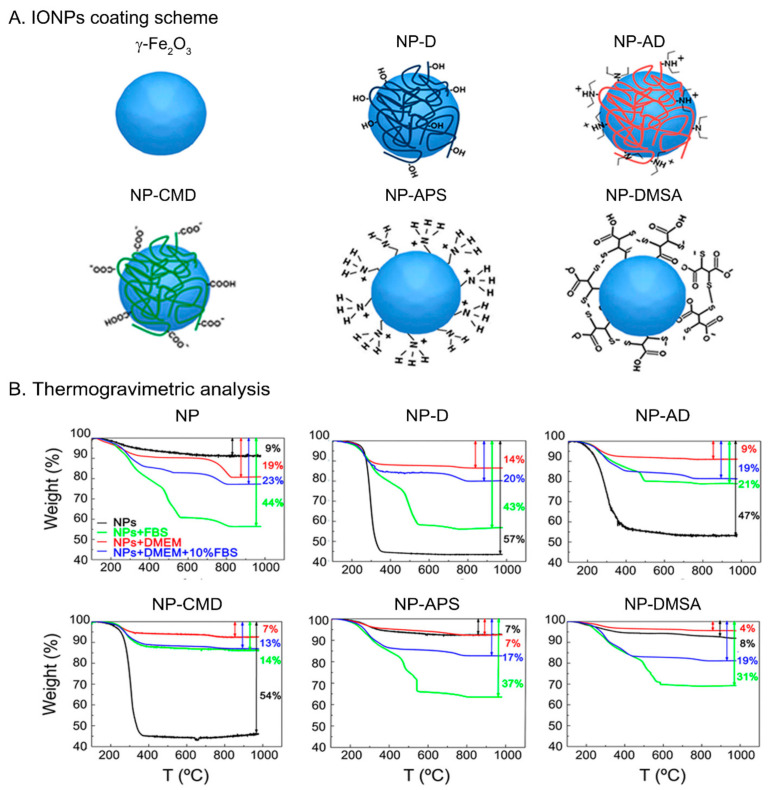
(**A**) Scheme illustrating the IONPs with the different coatings: NP (γ-Fe_2_O_3_), NP-D (dextran), NP-AD (amino-dextran), NP-CMD (carboxymethyl-dextran), NP-APS (aminopropyl-trietoxy silane), NP-DMSA (dimercaptosuccinic acid). (**B**) TGA measurements of IONPs suspended in different media, water (black), FBS (green), DMEM (red), and DMEM (10% FBS) (blue).

IONPs with a mean core size of 14.4 ± 3.7 nm, measured by transmission electron microscopy (TEM) and quasi-spherical shape (see Supporting Information Figure S1A–C) were prepared by the classical Massart’s method [35]. An oxidative acid treatment was performed in order to obtain γ-Fe_2_O_3_ NPs (Figure S1D,E) [36,46]. IONPs were named as follows, depending on the coating (Figure 1A): NP (naked), NP-D (dextran), NP-AD (amino-dextran), NP-CMD (carboxymethyl-dextran), NP-APS (aminopropyl-trietoxy silane), NP-DMSA (dimercaptosuccinic acid). Dextran derivatives are polysaccharides that provide steric stabilization to the inorganic core of the IONPs. In addition, electrostatic stabilization exists also in NP-CMD and NP-AD. By contrast, in the case of NP, NP-DMSA, and NP-APS, there is no steric hindrance protecting the IONPs (or it is negligible) and the stabilization is mainly due to electrostatic repulsion.

When NPs are dispersed in a liquid medium, they frequently undergo aggregation [47,48] as well as adsorption or binding to other solutes present in the medium [49,50,51]. Via DLS and TGA techniques, aggregation and adsorption processes can be accessed to provide: (i) *D_hyd_* values, obtained by DLS, which are strongly correlated to aggregate sizes; (ii) *ζ* potentials, which are related to the contribution of the electrostatic repulsion to the colloidal stability; and (iii) the weight loss, measured by TGA, which yields the amount of organic matter present in the material, i.e., of the coatings and the organic molecules present in culture media such as Dulbecco’s modified eagle’s medium (DMEM) or fetal bovine serum (FBS). The trend of IONPs aggregation in different media for samples NP, NP-APS, and NP-DMSA, evaluated through their *D_hyd_* values, can be sorted as follows: water < DMEM (10% FBS) < DMEM (Table 1). These samples are not stable in DMEM alone, where aggregate sizes larger than 1 μm were measured and precipitation of the particles was observed. Besides, very similar aggregate sizes were measured for NP-D and NP-CMD dispersed in the three media and a much higher *D_hyd_* value for NP-AD dispersed in DMEM + 10% FBS than in water or DMEM alone. It is known that the interaction of NPs with proteins can increase the *D_hyd_* either by causing aggregation or by simple adhesion. The later has been purposely employed for engineering NPs and, alone, cannot account for large increases in *D_hyd_* values similar to the ones observed here [52]. On the other hand, aggregation of NPs in protein-rich media has been suggested to rise the local concentration of superparamagnetic IONPs in mesenchymal stem cells. This feature must be controlled or monitored, since it can complicate cell tracking by MRI [53]. 

The *ζ* potential values measured in water (pH = 7.4) show that, as expected, NP-APS and NP-AD have high positive charges, NP-DMSA and NP-CMD large negative charges, NP-D is almost neutral, and the uncoated NP has a slightly negative value (Table 1). When IONPs are suspended in DMEM and DMEM + 10% FBS, NP-DMSA, and NP-CMD shift towards less negative *ζ* potential values while the initially positive NP-AD and NP-APS shift to less positive or even negative values. The reduction in *ζ* potential values in DMEM can be explained in terms of the interaction of the IONPs’ surfaces with the solutes present in the medium (amino acids, vitamins, glucose, and various inorganic ions), which eventually causes the charge shielding [54]. A similar effect over the *ζ* potential values can take place when the IONPs are suspended in DMEM + 10% FBS where IONPs are covered by plasma proteins [49]. NP-protein corona formation is known to produce such a shift of the charges [54], even from positive to negative values [55].

Considering *D_hyd_* and *ζ* potential values (Table 1) it is clear that DMEM causes strong reductions in the *ζ* potential values of all charged IONPs, causing aggregation and destabilizing them when they are mainly stabilized by electrostatic repulsion (NP, NP-APS, NP-DMSA) but with no increase of the aggregate size when they are coated with dextran derivatives. Thus, stabilization by steric hindrance is far more efficient than stabilization by electrostatic repulsion. In DMEM supplemented with 10% of FBS, the *ζ* potential values are also affected, but the aggregation of the particles is in general inhibited due to the presence of serum proteins in FBS that can adhere to the IONPs surfaces providing a great steric hindrance that stabilizes the particles [40].

To further understand these behaviors, weight losses were measured by TGA on powder samples prepared from the stock water dispersions and also after incubation in DMEM, DMEM + 10% FBS and FBS. In general, the larger the weight loss, the greater the amount of organic molecules per NP. The trend we observed (DMEM < DMEM + 10% FBS ≤ FBS; see Figure 1B) is coherent with a higher amount of proteins being attached to the IONPs’ surfaces as the concentration of proteins in the medium increases. When these results are compared with the TGA performed over samples without incubation in biological media, where only the initial coating accounts for the weight loss, a more complex behavior occurs. 

For the naked NP and NP-APS samples, the lowest weight loss is obtained when no incubation has been carried out, as expected, although for NP-APS there is almost no difference between IONPs from the water dispersion and from DMEM incubation. With NP-DMSA the weight loss is slightly higher for IONPs from the water dispersion than after incubation with DMEM, indicating that a partial stripping of the DMSA ligand from the surface caused by the DMEM medium has occurred. In addition, it cannot be discarded that other molecules present in DMEM become attached to the surface of the NPs. This ligand stripping is responsible, together with the electrostatic quenching of the surface charge caused by ions, of the aggregation and destabilization observed in NP, NP-APS, and NP-DMSA (see *D_hyd_* values from Table 1). If DMEM is supplemented with proteins (DMEM + 10% FBS, FBS), protein adhesion takes place, and the dispersion remains stable in spite of APS or DMSA stripping.

With dextran derivatives, the highest weight loss always occurs by far in the samples without incubation. Thus, the coating is removed to some extent even when it is composed of macromolecules. Despite this, the aggregate size is kept almost unchanged in DMEM, which means that after the partial removal of the coating in NP-D, NP-AD and NP-CMD in that medium, the remaining carbohydrates at the surface are enough to preserve the initial aggregate almost unchanged. Nevertheless, from our results, it is difficult to give a thorough explanation to the fact that the NP-AD has a larger aggregate size in DMEM + 10% FBS and further investigation would be needed to clarify this point [56,57].

These DLS and TGA results support that IONPs suspended in biological media experience a combination of two processes: (i) removal of the coating (in a greater or lesser extent) and (ii) protein adhesion (protein corona formation). Both of them contribute to the changes commonly observed in surface charge and *D_hyd_* values, including a higher aggregation in DMEM and aggregate stabilization in the presence of proteins. In addition, and importantly, they will influence the interaction of IONPs with the cellular membrane.

### 3.2. IONPs Uptake by the CMI Method

The internalization of IONPs into cells is strongly influenced by their coating and colloidal properties [58], but also by other parameters such as IONPs sterilization and suspension media [41]. In order to evaluate the role of the different coatings, we have used the same IONP core with the above five different coatings to assess their CMI-mediated internalization capabilities. We first resuspended the IONPs in DMEM supplemented with 10% of FBS and used a filter as the sterilization procedure. Surprisingly, after carrying out the CMI method, we observed from optical microscopy images of Prussian blue staining, that only two types of IONPs’ coatings (AD and DMSA) were able to cross the cellular membrane into the cytoplasm (Figure S2A). We speculated that these results might be due to their higher *D_hyd_* (AD: 561 nm; and DMSA: 335 nm) compared with the other IONPs.

**Figure 2:**
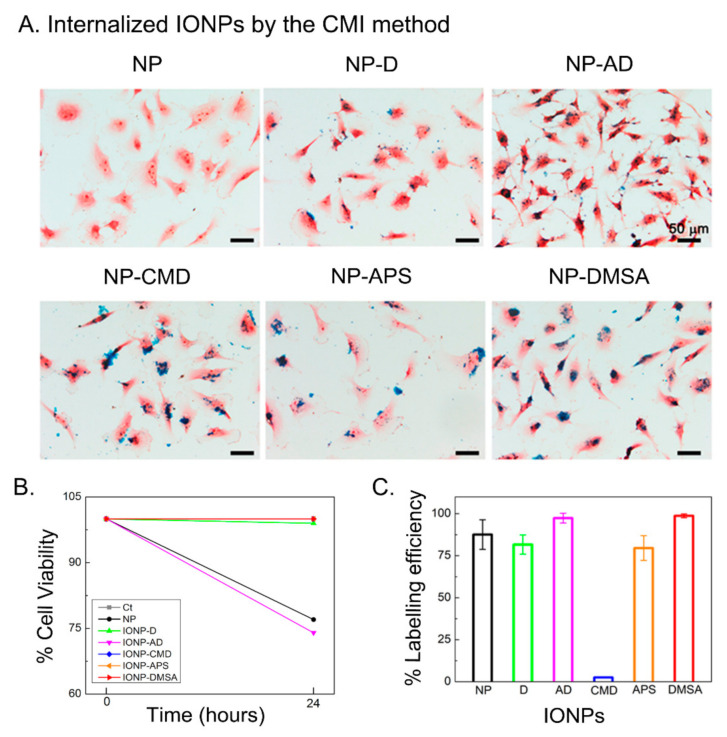
(**A**) Optical microscopy images of U373 cells after IONPs internalization through the CMI method followed by Prussian blue staining. (**B**) Cell viability obtained from resazurin assay. (**C**) IONPs labelling efficiency after CMI. Error bars represent standard deviations.

Accordingly, we tried out different concentrations of FBS (0% and 10%), which directly influence the *D_hyd_* (Table 1), and should have an impact on the uptake of IONPs by cells, once they reach the values observed for NP-AD and NP-DMSA. Higher speeds and times of incubations can produce additional aggregations and IONPs destabilization when they are under centrifugation forces [59]. Since the elimination of FBS in the media can increase the hydrodynamic size of IONPs (Table 1), the centrifugation time was reduced to 1 min, instead of 5 min, to avoid additional IONPs aggregations during CMI but still obtain high labelling efficiency [26]. The CMI method was performed using 1500 rpm, where the sedimentation and diffusion forces play an important role without producing any precipitation. Theoretical simulations and our experiments support there is no additional aggregation or IONPs destabilization under the CMI parameters used in this study [27]. Additionally, as filtering may retain part of the IONPs, we changed the sterilization protocol to UV irradiation in order to avoid the loss of IONPs. From Figure S2B, we observed that by simply changing the sterilization protocol we induced IONPs internalization using NP-AD, NP-APS, and NP-DMSA. Therefore, the concentration of FBS and the sterilization protocol altered the CMI-mediated internalization of all types of IONPs assayed, except those coated with CMD (Figure S2C). Depending on CMI parameters, Prussian blue images showed a possible IONPs aggregation on the cellular membrane surface. SEM and transmission electron microscopy (TEM) have been used to deeply understand if the interaction of IONPs with cells is external (aggregates on the surface), internal (IONPs uptake by cells), or both, internal and external interactions [59,60]. Previous studies used TEM to corroborate the internalization of IONPs by CMI method [26]. In addition, we observed the formation of agglomerates on top of the cellular membrane by SEM images, that were correlated with higher *D_hyd_*, for NP-AD (561 nm), NP-APS (1342 nm) and NP-DMSA (1636 nm) coatings (see SI and Figure S2D). Based on this and previous observations, we fine-tuned the FBS concentration and the type of sterilization protocol until we reached the values that yielded the best performance between CMI-mediated IONPs internalization and avoiding the formation of agglomerates. The best internalization results were obtained when naked NP, NP-D, NP-CMD, and NP-AD were resuspended in DMEM without FBS and using UV as sterilization procedure. Meanwhile, NP-APS (UV sterilization) and NP-DMSA (filtered) were suspended in DMEM with 10% of FBS (Figure 2A, Table S1).

Interestingly, Prussian blue images show that higher amounts of NP-DMSA and NP-AD arrive at the cell membrane and are, therefore, internalized (Figure 2A). The two main features of the IONPs studied here that can influence the variations observed in the internalization are the hydrodynamic diameter (related with the aggregate size) and the *ζ* potential. As discussed above, IONPs suspended in DMEM and DMEM + 10% FBS experience changes in both of them. The aggregate size will predictably be important for the internalization through the CMI method [26,27] and positive *ζ* potential values have been reported to enhance it when the classical internalization method by gravity is used [58,61,62,63,64]. Our results for CMD- and DMSA-coated NPs support a much higher influence of the aggregate size than of the *ζ* potential. Both are negative coatings, but NP-DMSA has a much larger aggregate size (335 nm) and a much higher labelling efficiency (98.7%) than NP-CMD (84 nm and 2.5%). Indeed, the labelling efficiency of NP-DMSA is similar to the one obtained with NP-AD (97.3%) and higher than the labelling efficiency with NP-APS (79.5%), both with positive coatings (Figure 2C). Therefore, we can conclude that when NPs are internalized using the CMI method, the aggregate size is the most critical feature (Table 1). The cell viability was studied by using Resazurin dye. This assay indicates no toxicity in most cases (Figure 2B), although naked NP and NP-AD presented 77% and 74% of cell viability, respectively. Probably due to small agglomeration in the case of naked NP or because of the presence of amino dextran groups for the NP-AD [65]. 

In summary, the suspension media chosen is an important parameter that influences the internalization of IONPs by modifying their colloidal properties. DMEM and DMEM supplemented with FBS cause variation in the hydrodynamic diameters (aggregate sizes) and *ζ* potential values affecting the internalization and labelling efficiency of IONPs by the CMI method. It is important to highlight that when trying to maximize the uptake of NPs by cells through the classical internalization method by gravity, the lowest aggregation degree is commonly preferred. However, when the centrifugation mediated internalization method is chosen, our results show that better labelling efficiencies are obtained with larger aggregates (provided that the dispersion is colloidally stable). The highest labelling efficiencies were observed for NP-DMSA and NP-AD. The former exhibited no toxicity, but the latter did. Therefore, DMSA coating was chosen to study the influence of core size in cell internalization and MRI contrast enhancement efficiency.

### 3.3. Influence of Core Size on IONPs Uptake by the CMI Method

**Figure 3:**
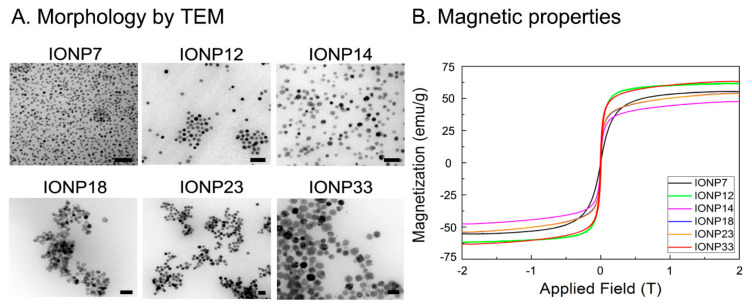
(**A**) Morphology characterization by TEM and (**B**) magnetic characterization by VSM of all IONPs with different core sizes. Scale bar: 50 nm.

After studying the effect of the coating using the same cores, we investigated the effect of the core size using the same coating of DMSA, which offered the best labelling efficiencies with no toxicity. Thus, IONPs with core sizes of 7, 12, 14, 18, 23 and 33 nm were synthesized by thermal decomposition. TEM micrographs with IONPs’ size and size distribution are shown in Figure 3A. The magnetic properties of these IONPs were studied by VSM measurements. Figure 3B shows the magnetization of IONPs with different core sizes as a function of the applied magnetic field. All IONPs presented a superparamagnetic behaviour. 

NPs were then coated with DMSA, through a ligand substitution procedure and the aggregate sizes were evaluated through their hydrodynamic diameters (Table S2). *D_hyd_* in different media follow the trend water < DMEM + 10% FBS < DMEM. As in the previous examples with different coatings, there is an increase in *D_hyd_* when IONPs are dispersed on DMEM, reaching aggregate sizes at the micron level. Again, the presence of FBS helps to partially stabilize the NPs and the aggregation is limited to sizes in the range 200–500 nm (Table S2). The lowest values of *D_hyd_* are obtained with IONP14 (212 nm) and IONP18 (223 nm), clearly differentiated from the rest, which present *D_hyd_* ≥ 300 nm. *ζ* potential values shift to less negative values the IONPs are dispersed in DMEM or DMEM + 10% FBS, which is again consistent with the results obtained with the different coatings.

**Figure 4:**
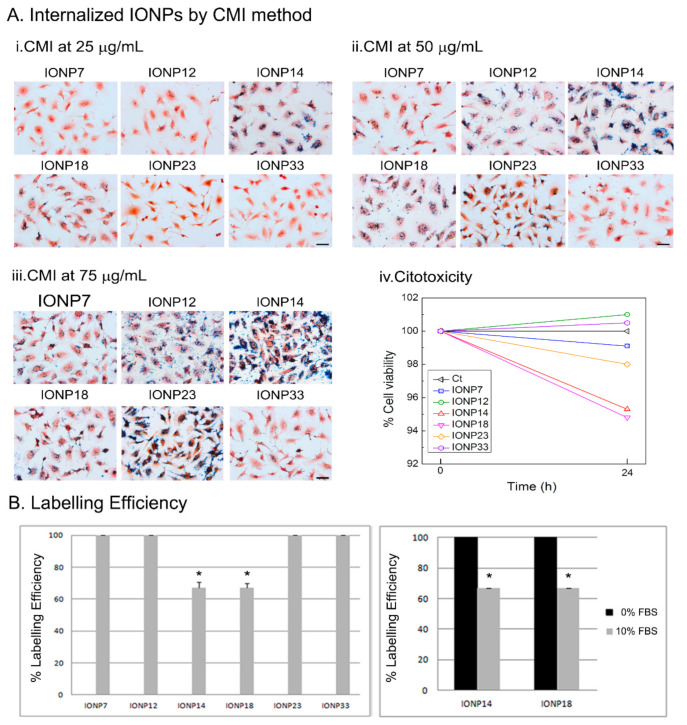
(**A**) Labelled cells by IONPs using CMI method. Optical images after Prussian blue staining of U373 cell line using different IONPs concentrations of (i) 25 μg/mL, (ii) 50 μg/mL, and (iii) 75 μg/mL and (iv) cell viability using 75 μg/mL of IONPs incubated after 24 h. (**B**) IONPs labelling on U373 cells at an initial concentration of 75 μg/mL using DMEM supplemented with 10% of FBS (left) or without FBS (right). A significant increase in labelling efficiency was obtained when IONPs were suspended in DMEM+0%FBS compared with DMEM supplemented with 10%FBS for IONP14 and IONP18.

With IONP14 and IONP18 suspended in DMEM 10% FBS, low internalizations by CMI were observed because of their small *D_hyd_*. Therefore, IONP14 and IONP18 were employed in DMEM only, while the remaining IONPs were suspended in DMEM + 10% FBS. Figure S3 shows the optical images after Prussian blue staining at different initial IONP23 concentrations. Agglomerates appeared on the cell surface concentrations using 100 and 125 µg/mL. Therefore, 25, 50 and 75 µg/mL of initial concentration were used to favour internalization. Figure 4A shows optical images of U373 cells incubated with IONPs at different concentrations using the CMI method and after Prussian blue staining. At 25 µg/mL, only IONP14 and IONP18 were internalized (Figure 4A) (i). At 50 µg/mL, only IONP7 and IONP23 were unable to undergo internalization (Figure 4A) (ii). Finally, at 75 µg/mL, IONPs underwent internalization and labelled the cells (Figure 4A) (iii). Resazurin assay indicated no toxicity using any of the IONPs by CMI after 24 h at an initial concentration of 75 µg/mL (Figure 4A) (iv).

The labelling efficiencies with U373 cells incubated at 75 μg/mL are almost 100% for all IONPs (Figure 4B). The labelling efficiencies for IONP14 and IONP18 in DMEM with and without FBS have been measured for comparison and to illustrate the influence of the colloidal changes induced by the cell culture media. With both types of IONPs, labelling efficiencies of ≃100% are reached in DMEM, while in DMEM + 10% FBS the labelling efficiencies drop dramatically below 70% (Figure 4B). These data corroborate that higher hydrodynamic diameters could induce an increase in cell labelling using the CMI methodology.

### 3.4. MRI Contrast Enhancement In Vitro

**Figure 5:**
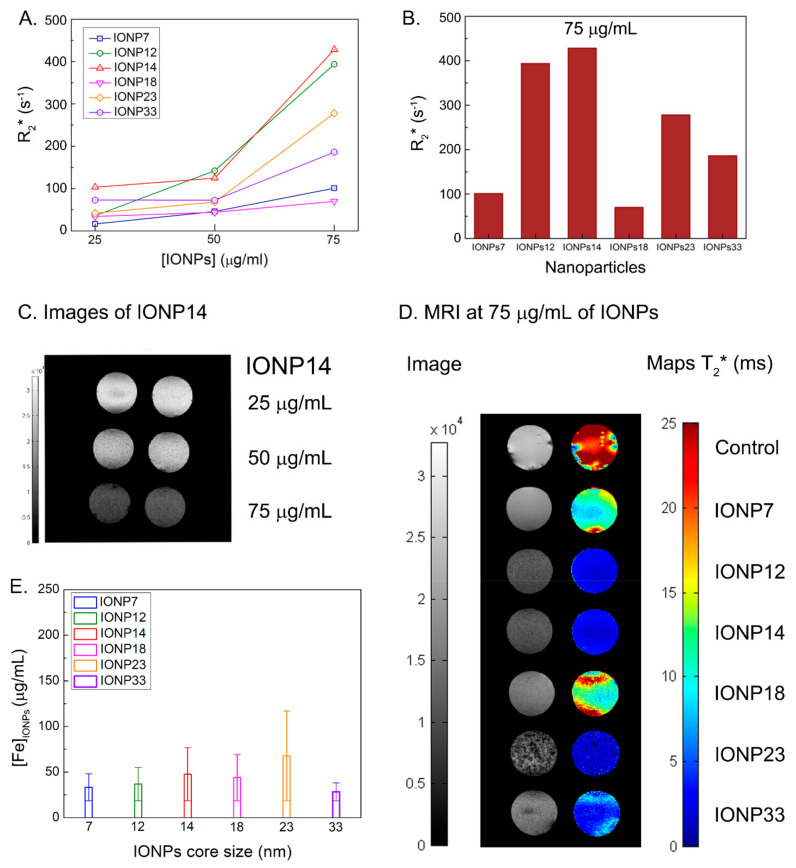
(**A**) *R*_2_^*^ values at three different IONPs concentrations for all the core NPs sizes. (**B**) *R*_2_^*^ values of all IONPs at a concentration of 75 μg/mL. See the highest values for IONP14 and IONP12. (**C**) Duplicate contrast images of all IONPs at an initial concentration of 75 μg/mL of IONP14 show a better contrast when increasing the concentration of IONPs. (**D**) Contrast and *T*_2_^*^ maps of all IONPs at a concentration of 75 μg/mL. (**E**) Intracellular iron concentration after CMI method measured by Prussian blue staining images using 75 μg/mL of IONPs.

NPs could be treated as positive or negative contrast agents depending on their particle size, coating and magnetic properties [30,66]. Positive contrast agents are made of NPs with small core sizes (<5 nm), coatings that provide good stability with hydrodynamic sizes between 10–20 nm and paramagnetic properties. These parameters support the *T*_1_ effect [67]. Since in this study we used superparamagnetic IONPs, with core sizes between 7–23 nm and higher hydrodynamic sizes, they could be treated as a negative contrast agent which reduces *T*_2_ signals by shortening the *T*_2_ relaxation time [30,67]. After incubating the cells with IONPs at concentrations of 25, 50 and 75 μg/mL, using the CMI method, the relaxation rates *R*_1_, *R*_2_, and *R*_2_^*^ were calculated from the measured relaxation times *T*_1_, *T*_2_, and *T*_2_^*^. These values, together with the *R*_2_/*R*_1_ ratio, are collected in Table 2. Using a concentration of 75 µg/mL, IONP12 and IONP14 developed the higher values of *R*_2_, *R*_2_^*^ and *R*_2_/*R*_1_ (Figure 5 and Figure S4).

Since these IONPs are known as efficient *R*_2_ or *R*_2_^*^ (which takes into account *R*_2_ and magnetic field inhomogeneities) contrast agents, we are going to focus on these results. Figure 5A shows how *R*_2_^*^ values increase with the concentration (of incubation) of all IONPs. At 75 μg/mL it is clear that IONP14 and IONP12 present the topmost *R*_2_^*^ values, 428 s^−1^ and 394 s^−1^, respectively (Figure 5B and Table 1).

There have been many studies devoted to characterizing the relaxation rates of IONPs [68,69]. However, there are fewer examples dealing with relaxation rates of IONPs incubated with cells. For instance, Brisset et al. used 4.7 and 7 T scanners to study the relaxation rates of two types of IONPs: Ferumoxtran-10 (Sinerem^®^, in the past commercialized as contrast agent by Guerbet) and negatively charged IONPs with a mean core size of 8.7 nm [70]. With the same field as the one used in our experiments (7 T), they observed the best internalization and highest contrast in cells (10^6^ cells) with the negatively charged IONPs, with *R*_2_^*^ values up to 200 s^−1^ at the highest concentration. That *R*_2_^*^ is above the maximum one that we have measured with IONP7 (101 s^−1^) and well below the measured *R*_2_^*^ of IONP12 (394 s^−1^) and IONP14 (428 s^−1^). In another work, Zhang et al. studied the relaxation rates of DMSA-coated IONPs functionalized with methotrexate and encapsulated with the hepatitis B virus core antigen using a 7 T scanner [71]. The core size of these NPs was (11.7 ± 1.6) nm and the concentrations used for the incubation with cells were in the range [Fe] ≃ 200–3000 µg/mL. They tested 10^7^ cells and the highest *R*_2_ value that they observed was 70 s^−1^, obtained at the highest concentration of 3000 µg/mL IONPs. However, our experiments for IONP14 presented a higher *R*_2_ value (84 s^−1^) using a concentration of NPs 40 times lower than in this study. A recent study using IONPs coated with an amino alcohol derivate of glucose reached the highest labelling and intracellular iron content using an initial concentration of 50 µg/mL. The *R*_2_ value from a 7 T magnetic resonance image was close to 40 s^−1^ but they also observed a decrease in cellular viability using 50 µg/mL and needed to reduce to 37.5 µg/mL for the rest of their study (*R*_2_ ≃ 20 s^−1^) [72]. This value was below our best relaxation rate (Table 2) and highlights the efficiency of the internalization of IONPs in cells using the CMI method for excellent labelling and viability. 

Characterizing the colloidal properties of IONPs is relevant for optimizing their performance and D_hyd_ ≥ 300 nm are the most appropriate when applying CMI. Using this methodology, we were able to have faster labelling and internalization of IONPs in cells than conventional methods employing gravity [26,27]. We observed that *R*_2_^*^ increases while incrementing the core size from 7 to 12 nm, achieving the maximum value for a core of 14 nm. Further pushing those sizes to 18 nm, 23 nm and 33 nm will produce a decay of the *R*_2_^*^ value. The colloidal properties will enhance the labelling of IONPs, but MRI responses have a clear relation with the IONPs core showing excellent results when using 12–14 nm core sizes. The darker signal due to the IONPs contrast can be appreciated in Figure 5C, where darker images are obtained as the concentration of IONP14 increases. In general, IONPs with core sizes of 12 and 14 nm produce better contrasts on the MRI maps (Figure 5D left) and lower *T*_2_^*^ values (Figure 5C right) than IONPs with larger or smaller cores and, therefore, they are more suitable NPs for U373 cell labelling and MRI detection.

To determine if the higher R_2_* values are due to a higher intracellular iron after CMI method, we calculated the iron concentration after CMI by Prussian blue staining. The highest amount of IONPs inside of the cell was obtained using IONPs with a core size of 23 nm with 67.8 µg/mL (Figure 5E). The concentration for IONP12 and IONP14 was 36.9 µg/mL and 47.6 µg/mL, respectively. The upmost contrast enhancement found by MRI was obtained using IONPs with a 14 nm of core size, coated with DMSA, at an initial concentration of 75 μg/mL. These IONPs provide a new experimental source for MRI in cancer diagnosis. Therefore, the high *T*_2_ contrast enhancement obtained for both IONPs, IONP12 and IONP14, must be due to a combination of parameters: colloidal properties based on core size and coating and high labelling efficiency using the CMI method. Controlling these NPs synthesis parameters and their uptake methodology will provide the possibility of the modification for future agent labels for improving the relaxation rates for MRI applications. 

## 4. Conclusions

The characterization of colloidal properties of IONPs and their interaction with biofluids is important in order to study the labelling ability. In conclusion, we found that IONPs suspended in biological media experience a partial removal of the coating and adhesion of proteins and other molecules. The FBS concentration alters the CMI-mediated internalization of all types of IONPs assayed, except in those coated with CMD. Interestingly, using CMI with IONPs coated with small molecules, as DMSA, the cells present the highest labelling efficiency. The difference in the core size did not change the colloidal properties for CMI methodology. Using an initial concentration of 75 μg/mL of IONPs with a core size of 14 nm and coated with DMSA we obtained the highest relaxation rate *R*_2_^*^, 422 s^−1^ and a coefficient *R*_2_/*R*_1_ of 144.7 by MRI measurements. These results showed that a good contrast will require using a core size range between 12–14 nm coated with DMSA in order to achieve selected colloidal properties to obtain greater labelling via the CMI methodology. Moreover, these results are in good agreement with the in-silico predictions of our previous work [27], where IONPs with DMSA exhibited strong flux changes towards cells in very short times when CMI was employed. This study opens the opportunity to use this new NP-based technology for improving the contrast in MRI. It will also be useful in other biomedical applications that need improvements in labelling cells for treatments in different diseases as cancer or in the regenerative medicine field.

## Figures and Tables

**Table 1:** Mean hydrodynamic diameters (with PDI in parentheses) and zeta potential values of IONPs suspended in water, DMEM cell culture medium and DMEM supplemented with FBS (10%).

IONPs	*D_hyd_* (nm)			*ζ* Potential (mV)		
Coating	Water	DMEM	DMEM +10%FBS	Water	DMEM	DMEM +10%FBS
NP	124 (0.1)	1347 (0.3)	137 (0.2)	−8	−8	13
NP-D	85 (0.2)	132 (0.3)	128 (0.2)	−0.5	−4	−6
NP-AD	81 (0.2)	76 (0.3)	561 (0.3)	37	10	−7
NP-CMD	84 (0.4)	84 (0.1)	79 (0.2)	−33	−18	−18
NP-APD	166 (0.6)	1342 (0.3)	177 (0.2)	27	−6	−1
NP-DMSA	124 (0.1)	1636 (0.2)	335 (0.3)	−26	−13	−12

**Table 2:** Relaxation rate values, *R*_1_, *R*_2_, and *R*_2_^*^ and coefficient *R*_2_/*R*_1_ values from MRI at different IONPs initial concentrations of 25, 50 and 75 μg/mL.

Samples	[IONPs] (μg/mL)	*R*_1_ (s^−1^)	*R*_2_ (s^−1^)	*R*_2_^*^ (s^−1^)	*R*_1_/*R*_2_
Control	50.000 cells	0.4	10.1	29.7	26.2
IONP7	25	0.5	8.9	16.4	17.7
	50	0.4	8.8	45.9	20.4
	75	0.3	8.6	101.0	29.3
IONP12	25	0.2	7.4	35.8	32.3
	50	0.5	22.8	142.3	46.4
	75	0.7	58.0	393.7	88.4
IONP14	25	0.4	13.6	103.3	33.0
	50	0.4	21.9	124.8	50.5
	75	0.6	83.9	428.3	144.7
IONP18	25	0.5	11.5	34.3	22.1
	50	0.5	12.7	43.9	26.6
	75	0.3	12.8	69.9	43.3
IONP23	25	0.3	10.6	42.0	31.6
	50	0.4	12.6	68.2	35.2
	75	0.4	32.9	278.0	80.5
IONP33	25	0.4	9.7	72.9	23.9
	50	0.4	13.2	72.5	34.2
	75	0.3	19.5	186.2	62.2

## Supplementary Materials

The following are available online at https://www.mdpi.com/article/10.3390/nano11112888/s1, Figure S1: (A–C) Selected TEM micrographs and histogram showing the size distribution of IONPs. (D) XRD pattern and diffraction maxima from maghemite nanoparticles. (E) VSM measurements for the different coated IONPs. Key words: NP (naked), NP-D (dextran), NP-AD (amino-dextran), NP-CMD (carboxymethyl-dextran), NP-APS (aminopropyl-trietoxy silane), and NP-DMSA (dimercaptosuccinic acid). Figure S2: Optical microscopy images of U373 cells after Prussian blue staining following: (A) filtered IONPs suspended in DMEM with 10% FBS, (B) un-filtered IONPs suspended in DMEM with 10% of FBS, and (C) un-filtered IONPs in DMEM at 0% of FBS. (D) Scanning electron microscopy of a control U373 cells, filtered NP-AD on DMEM 10% FBS, un-filtered NP-APS and NP-DMS on DMEM 0% FBS. White arrows show IONPs on the cell membrane surface. Scale bars: black = 50 µm, white = 25 µm. Table S1: Colloidal properties of IONPs from Table 1, after CMI improvements. Table S2: Mean hydrodynamic diameters (with PDI in parentheses) and zeta potential values of IONPs with different size core suspended in water, DMEM cell culture medium and DMEM supplemented with FBS (10%). Figure S3: Prussian blue optical images of IONP23 on U373 cells at different concentrations, 25, 50, 75, 100 and 125 μg/mL. Scale bar: 50 μm. Figure S4: contrast images and maps of (A) *T*_1_, (B) *T*_2_ at an initial concentration of 25 μg/mL, 50 μg/mL and 75 μg/mL for all IONPs. (C) (left) *R*_1_ (up) and *R*_2_ (down) values for all IONPs at different concentrations. (C) (right) values of *R*_1_ (up) and *R*_2_ (down) using 75 μg/mL for all IONPs. (D) Contrast and maps of *T*_2_^*^ of all IONPs using 25 μg/mL, 50 μg/mL and 75 μg/mL initial concentration.
