# Supplementary material for: Influence of Coating and Size of Magnetic Nanoparticles on Cellular Uptake for In Vitro MRI
Source: Nanomaterials (Basel). 2021 Oct 28;11(11):2888. doi: 10.3390/nano11112888 (PMC8625532; doi:10.3390/nano11112888)
Supplement: Supplementary file 1 [file nanomaterials-11-02888-s001.zip › nanomaterials-1407792-supplementary update.pdf]

nanomaterials

MDPI

Supplementary Information

Influence of Coating and Size of Magnetic Nanoparticles on Cellular Uptake for In Vitro MRI

Belén Cortés-Llanos [1,2,3], Sandra M. Ocampo [1], Leonor de la Cueva [1], Gabriel F. Calvo [4], Juan Belmonte-Beita [4], Lucas Pérez [1,3], Gorka Salas [1,*], Ángel Ayuso-Sacido [5,6,7,*]

[1] IMDEA Nanoscience, Ciudad Universitaria de Cantoblanco, 28049 Madrid, Spain; belencortes@ucm.es (B.C.-L.); samioca75@gmail.com (S.M.O.); ldelacueva@euro-funding.com (L.d.l.C.); lucas.perez@ucm.es (L.P.)
[2] Department of Bioengineering, University of Washington, Seattle, WA 98195, USA
[3] Department of Materials Physics, Complutense University of Madrid, 28040 Madrid, Spain
[4] MOLAB-Mathematical Oncology Laboratory, Department of Mathematics, Universidad de Castilla-La Mancha, 13071 Ciudad Real, Spain; gabriel.fernandez@uclm.es (G.F.C.) and juan.belmonte@uclm.es (J.B.-B.)
[5] Brain Tumor Laboratory, Fundación Vithas, Grupo Hospitales Vithas, 28043 Madrid, Spain
[6] Faculty of Experimental Sciences, Universidad Francisco de Vitoria, 28223 Madrid, Spain
[7] Faculty of Medicine, Universidad Francisco de Vitoria, 28223 Madrid, Spain
* Correspondence: gorka.salas@imdea.org (G.S.); ayusosacido@gmail.com (A.A.-S.); Tel.: +34-912-998-850 (G.S.); +34-686-966-904 (A.A.-S.)

IONPs characterization

XRD and VSM measurements

The diffraction maxima (Figure S1D) have been indexed as 220, 311, 400, 422, 511 and 440 reflections corresponding all of them to the maghemite phase (ICDD 00-039-1346). Almost identical M_S values in the range 74–79 emu/g, typical of maghemite, were measured for all samples (Figure S1E).

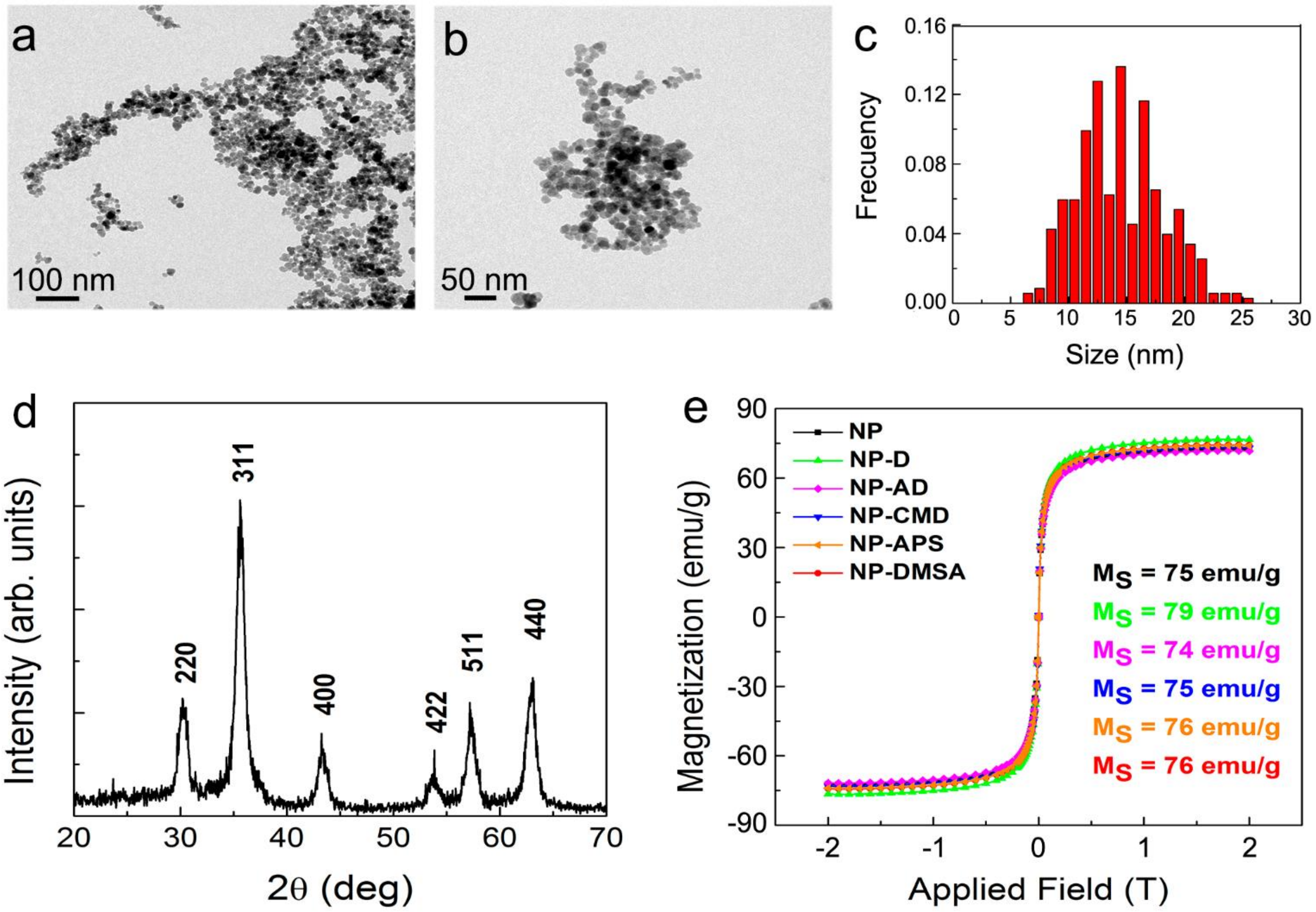

Figure S1. a–**c**) Selected TEM micrographs and histogram showing the size distribution of IONPs. **d**) XRD pattern and diffraction maxima from maghemite nanoparticles. **e**) VSM measurements for the different coated IONPs. Key words: NP (naked), NP-D (dextran), NP-AD (amino-dextran), NP-CMD (carboxymethyl-dextran), NP-APS (aminopropyl-trietoxy silane), and NP-DMSA (dimercaptosuccinic acid).

IONPs uptake by the CMI method

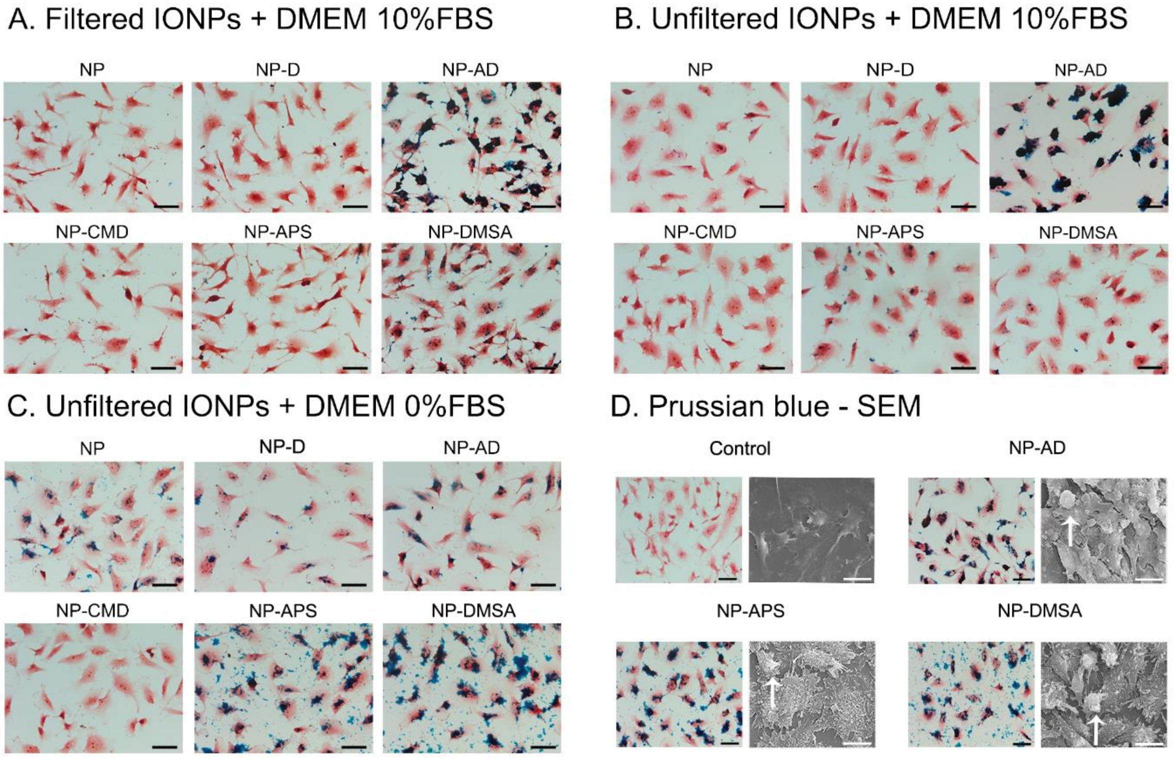

Figure S2. Optical microscopy images of U373 cells after Prussian blue staining following **A**) Filtered IONPs suspended in DMEM with 10% FBS, **B**) Un-filtered IONPs suspended in DMEM with 10% of FBS, **C**) Un-filtered IONPs in DMEM at 0% of FBS. **D**) Scanning electron microscopy of a control U373 cells, filtered NP-AD on DMEM 10% FBS, un-filtered NP-APS and NP-DMS on DMEM 0% FBS. White arrows show IONPs on the cell membrane surface. Scale bars: black = 50 μm, white = 25 μm.

Colloidal properties

Table S1. Colloidal properties of IONPs Table 1, after CMI improvements.

IONPs	D_{hyd} (nm)	ζ potential (mV)	Labelling (%)
NP	1347 (0.3)	−8	97.6 ± 8.8
NP-D	132 (0.3)	−4	81.6 ± 5.7
NP-AD	76 (0.3)	10	97.3 ± 2.9
NP-CMD	84 (0.1)	−18	2.5 ± 0.4
NP-APS	177 (0.2)	−1	79.5 ± 7.4
NP-DMSA	335 (0.3)	−12	98.7 ± 1.2

Table S2. Mean hydrodynamic diameters (with PDI in parentheses) and zeta potential values of IONPs with different size core suspended in water, DMEM cell culture medium and DMEM supplemented with FBS (10%).

IONPs	D_{hyd} (nm)			ζ potential (mV)		
	Water	DMEM	DMEM+10%FBS	Water	DMEM	DMEM+10%FBS
IONP7	53 (0.1)	958 (0.3)	376 (0.3)	−43	−15	−11
IONP12	78 (0.3)	1014 (0.2)	357 (0.3)	−41	−10	−12
IONP14	49 (0.4)	1002 (0.7)	212 (0.3)	−37	−22	−12
IONP18	44 (0.2)	820 (0.2)	223 (0.4)	−37	−22	−12
IONP23	45 (0.2)	1946 (0.2)	424 (0.4)	−43	−16	−12
IONP33	85 (0.3)	834 (0.3)	350 (0.3)	−42	−14	−11

Figure S3 shows the optical images after Prussian blue staining at different initial IONP23 concentrations. With high initial concentrations of 100 and 125 μg/mL, agglomerates appeared on the cell surface, so 25, 50 and 75 μg/mL of initial concentration were used to favour internalization.

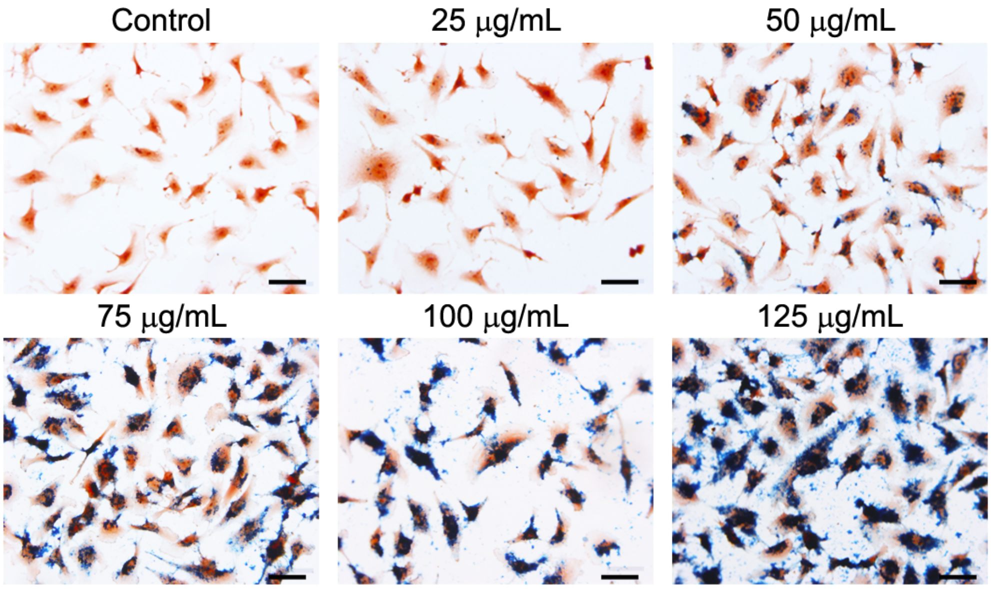

Figure S3. Prussian blue optical images of IONP23 on U373 cells at different concentrations, 25, 50, 75, 100 and 125 μg/mL. Scale bar: 50 μm.

MRI Contrast Enhancement In Vitro

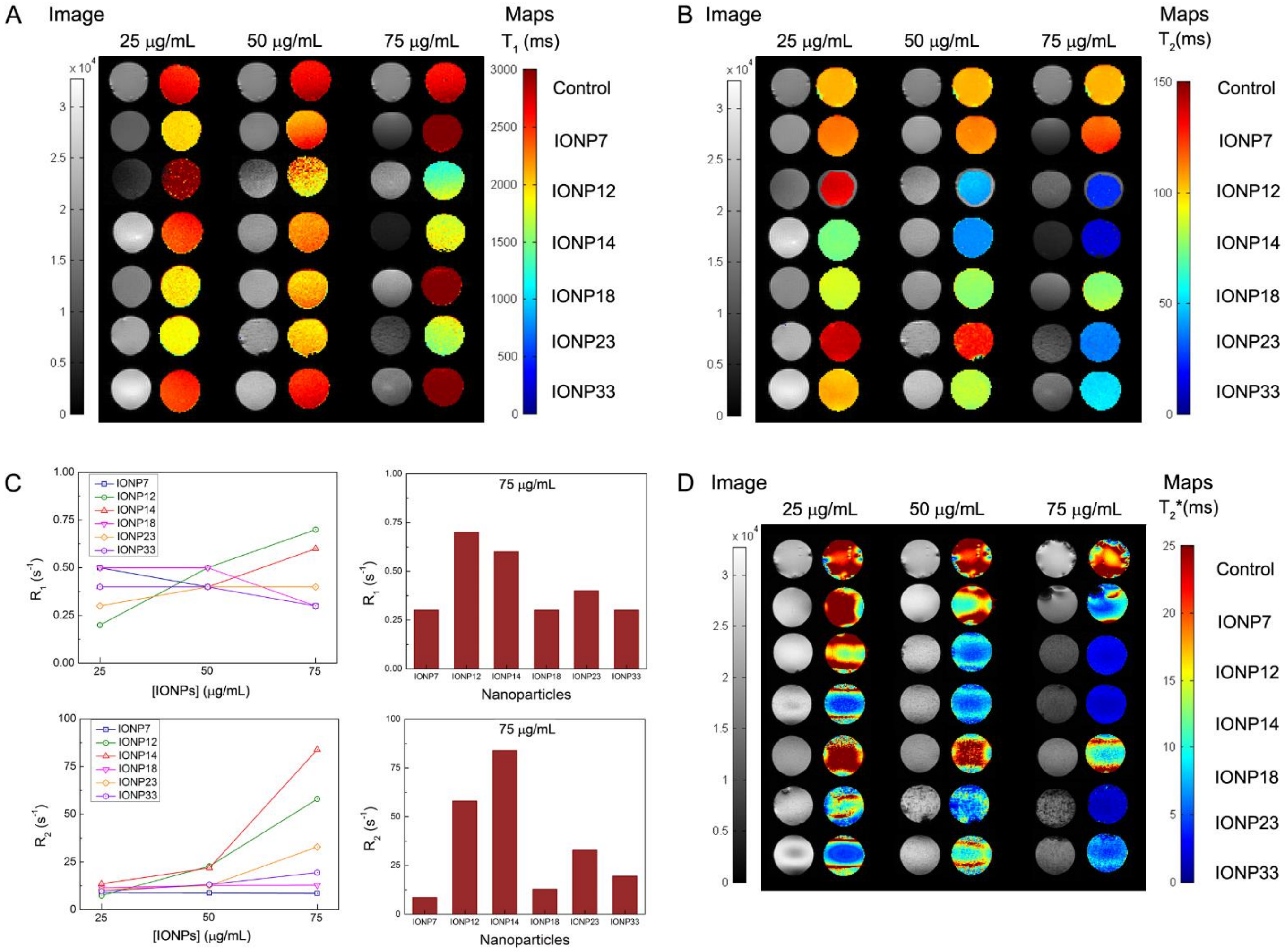

Figure S4. Contrast images and maps of **A**) T_1, **B**) T_2 at an initial concentration of 25 µg/mL, 50 µg/mL and 75 µg/mL for all IONPs. **C**, left) R_1 (up) and R_2 (down) values for all IONPs at different concentrations. **C**, right) Values of R_1 (up) and R_2 (down) using 75 µg/mL for all IONPs. **D**) Contrast and maps of T_2^* of all IONPs using 25 µg/mL, 50 µg/mL and 75 µg/mL initial concentrations.
